# Relationship between depression, anxiety, and perceived stress in health professionals and their perceptions about the quality of the health services in the context of COVID‐19 pandemic

**DOI:** 10.1002/brb3.2816

**Published:** 2022-11-25

**Authors:** Adriel Olórtegui‐Yzú, Johann M. Vega‐Dienstmaier, Alberto Fernández‐Arana

**Affiliations:** ^1^ Universidad Nacional Mayor de San Marcos Facultad de Medicina de San Fernando Lima Perú; ^2^ Instituto Nacional Cardiovascular—INCOR—EsSalud Lima Perú

**Keywords:** COVID‐19, health professionals, health services, mental health, perception

## Abstract

**Background:**

The health emergency caused by COVID‐19 revealed the shortcomings of health services (HS), but little is known about how this has impacted the mental health of health professionals (HP).

**Methods:**

Data were collected through an online survey administered to HP in Lima (Peru) between May and July 2020. Instruments were applied to evaluate anxiety, depression, perceived stress (PS), and perceived quality of health services (PQHS).

**Results:**

A total of 507 HP completed the survey. In the multivariate analysis, younger age and female gender were related to anxiety, depression, and PS (all with *p* < .001). The most relevant unfavorable PQHS associated with anxiety were competence of other HP to care for HP if infected (*p* = .002) and support for HP or their families in the event of becoming infected (*p* = .001); the most relevant unfavorable PHQS associated with depression were equipment to care for HP and their families if infected (*p* = .003); support for HP or their families if infected (*p* < .001); fear of HP and/or family members being infected or dying (*p* = .006); and HP’ recognition of their competencies (*p* < .001); and the most relevant unfavorable PHQS associated with PS were support for HP or their families if infected (*p* < .001) and instability of knowledge (*p* = .027).

**Conclusions:**

There was an association between impaired mental health and PQHS scores among HP. This study shows the need for HP to express their concerns about how HS are supporting their safety and that of their family during health emergencies.

## Graphical abstract

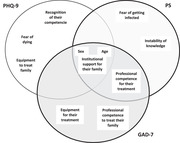

## 1 INTRODUCTION

The health emergency generated by the COVID‐19 pandemic put the solvency of health services to the test, not only in their ability to face the growing demand for patient care but also with regard to the availability of health professionals (HP), technological means for diagnosis and treatment, and medical supplies that would allow adequate care for patients and optimal working conditions for HP (Chen et al., 2020; Organización Panamericana de la Salud, 2022).

Due to the above, it is important to describe some peculiarities in relation to how the COVID‐19 pandemic has developed in Peru and its impact on HP. At the time of preparing this article, there were 113,550 HP infected by COVID‐19, among whom 2250 died, with Peru ranking third‐highest for HP mortality in the Americas (World Health Organization, 2021a, 2021b). Notably, Peru was one of the first countries to implement the health recommendations issued by the World Health Organization (WHO) to protect HP against the virus. For this reason, the apparent lack of efficacy of these strategies is striking (Cuba, 2021; Díaz‐Cassou et al., 2020). One explanation for this lack of efficacy is that in Peru, as in all countries of the Americas, HP had to face the pandemic under exceptional conditions: an increase in workload, lack of medications or specific vaccines, and lack of personal protective equipment and the feeling of not receiving sufficient support from health authorities (Shanafelt et al., 2020).

These working conditions, added to the impact of the pandemic on the quality of life of HP, could explain why when faced with COVID‐19 on the front line, the mental health of HP was affected (Fernández‐Arana et al., 2022; Lozano‐Vargas, 2020; Pearman et al., 2020; Siddiqui et al., 2021; Vizheh et al., 2020), diminishing their capacity for clinical understanding and/or their decision‐making skills (Young et al., 2021). Therefore, we must consider the COVID‐19 pandemic as a potent and persistent stressor and that its effects on the mental health of HP depended on, among other factors, the discomfort experienced as a result of poor working conditions and feelings of abandonment by their governments associated with precarious health services (HS) that failed in their duty to protect them (*Caring for people who care: supporting health workers during the COVID 19 pandemic—eClinicalMedicine*, s. f.; Charney et al., 2020; Lin et al., 2020; Organización Panamericana de la Salud, 2022).

The objective of this study was to evaluate whether there is a relationship between mental health problems (anxiety, depression, and perceived stress (PS) among HP in Lima (Peru) and their perceptions about the quality of health services (PQHS) in the context of the COVID‐19 pandemic.

## 2 MATERIALS AND METHODS

This was a cross‐sectional study that included 507 HP without a previous diagnosis of COVID‐19 from Lima and surrounding areas in Peru. The selection of professionals was by invitation. The sample size was calculated using Epidat v 3.1 (Consellería de Sanidade—Servizo Galego de Saúde, s. f.). Parameters for estimation were maximum variability (*p* = .5), significance of 0.005 and accuracy of 5.0%; also, we considered a 10% of data loss. Finally, the minimum sample required was 430 HP. The professions included physicians, nurses, midwives, medical technologists, nutritionists, pharmaceutical chemists, nursing technicians, psychologists and biologists. Regarding first‐line care, no professional distinction was made because due to the lack of personnel for the identification and care of patients with COVID‐19, health institutions assigned work directly or indirectly to all available HP.

The personal, work, health and mental health service variables were measured through a questionnaire developed with Google Forms® and distributed through the internet during the months of May and June 2020.

The Generalized Anxiety Disorder 7 (GAD‐7) scale was used to measure anxiety symptoms (Zhong et al., 2015); the Patient Health Questionnaire‐9 (PHQ‐9) was used to measure the level of depression (Villarreal‐Zegarra et al., 2019); and the Global Perceived Stress Scale, version 13 (EPGE‐13) was used to evaluate PS (Cohen et al., 1983; Guzmán‐Yacaman & Reyes‐Bossio, 2018). All these instruments have been psychometrically evaluated in Peru.

PQHS were identified through 20 questions, corresponding to 20 dichotomous variables (“favorable perception” = 1 or “unfavorable perception” = 0). This questionnaire was developed by the authors based on research from other studies on this topic (*Caring for people who care: supporting health workers during the COVID 19 pandemic—eClinicalMedicine*, s. f.; Charney et al., 2020; Lin et al., 2020; Organización Panamericana de la Salud, 2022). The correlation between the variables and perceptions can be seen in Table 1.

**TABLE 1:** Grouping and assignment of variables of PQHS of HP in the context of the pandemic

Direct variable	Statement
Empathy	1. Health authorities recognize and understand my concerns.
Recognition of competencies	2. Health authorities pay attention to my experience during health emergencies.
Protection	3. I receive the protective equipment appropriate for this pandemic.
Early personal diagnosis	4. I have quick access to diagnostic systems to know if I am infected by the virus.
Early family diagnosis	5. I have quick access to diagnostic systems to know if my family is infected with the virus.
Logistical support	6. I have the necessary equipment to care for infected patients.
Professional competence for their treatment	7. If I were infected, I would receive adequate care from my colleagues because they are trained.
Equipment for treatment	8. If I were infected, I would receive adequate care from the hospital because it has adequate equipment.
Professional competence for their family	9. If a family member were infected, they would receive adequate care because my colleagues are trained.
Equipment for their family	10. If a family member were infected, they would receive adequate care from the hospital because they have adequate equipment.
Fear of getting infected	11. I am afraid of getting infected.
Fear of infecting the family	12. I am afraid of infecting my loved ones.
Fear of dying	13. If I were infected, I would probably die.
Fear of a family member dying	14. If a family member was infected, he/she would probably die.
Role change	15. I am concerned about being in an area of care that is not my specialty.
Instability of knowledge	16. I am concerned that information and procedures change day by day.
Job burnout	17. The work is strenuous and does not allow me to be totally effective.
Concern for the family	18. I cannot stop thinking about my family when I am at work, and I have a hard time concentrating.
Institutional support for personal and family infection	19. If I got sick, I would receive support from the health authorities for me and my family.
Optimism	20. This pandemic will pass soon, and everything will change for the better.

### 2.1 Data analysis

Summary statistics were prepared, allowing an analysis of the characteristics of the sample and the behavior of the study variables. Then, using heteroscedastic linear regression, the relationship between the mental health variables (anxiety, depression, and PS) and each of the PQHS variables concerning the pandemic was explored. First, a bivariate analysis was performed to identify significant associations between PQHS scores and mental health variables. Those that were statistically significant were incorporated into the multivariate analysis together with the variables age and sex. From this analysis, a model was built that incorporated the independent variables one by one, basing the selection of the model on Akaike and Bayesian information criteria.

### 2.2 Ethical aspects

The study procedures were carried out in accordance with the ethical standards for research in humans. The study was approved by the Research Ethics Committee of the “Carlos Alberto Peschiera Carrillo” National Cardiovascular Institute—INCOR—EsSalud.

## 3 RESULTS

This study is part of the Mental Health of Healthcare Workers project, which was conducted during the pandemic in the city of Lima, Peru, in the months of May and June 2020. The project consisted of a survey to measure aspects of mental health related to the context of the pandemic that allowed an exploration of the influence of the pandemic context on the mental health of HP; the results were published at the end of 2021 (Fernández‐Arana et al., 2022).

The survey was answered by 507 HP, whose average age was 49.5 years (minimum of 23 years and maximum of 87 years). Among the participants, 62.7% were women, with a mean age of 47.7 years; for men, the mean age was 52.5 years (*p* < .05).

The response rate was highest for the groups of doctors and nurses, 56.8% and 15.0%, respectively, followed by obstetric professionals (11.6%). These 3 groups represented 88.4% of the total number of respondents. Professionals living with someone or married accounted for 59.5% of the participants. A total of 38.3% of respondents worked directly with patients with COVID‐19.

Regarding the PQHS of the HP not infected by COVID‐19, the 2 most frequently reported items were “fear of infecting their family and themselves,” with 92.5% and 89.0%, respectively. These were followed by the items “instability of knowledge” and “fear of a family member dying,” with 80.5% and 72.0%, respectively; “job burnout” also stands out, reaching 69.0% (see Table 2).

**TABLE 2:** Frequency of PQHS variables among HP in the context of the pandemic

Perception assessed	Negative		Positive		Total
	No.	%	No.	%	No.
12. Fear of infecting the family	469	92.5	38	7.5	507
11. Fear of getting infected	451	89.0	56	11.0	507
16. Instability of knowledge	408	80.5	99	19.5	507
14. Fear of a family member dying	365	72.0	142	28.0	507
17. Job burnout	350	69.0	157	31.0	507
5. Early family diagnosis	312	61.5	195	38.5	507
13. Fear of dying	309	60.9	198	39.1	507
15. Role change	304	60.0	203	40.0	507
18. Concern for family	293	57.8	214	42.2	507
18. Institutional support for their family	255	50.3	252	49.7	507
4. Early diagnosis	242	47.7	265	52.3	507
10. Equipment for their family	240	47.3	267	52.7	507
8. Equipment for treatment	218	43.0	289	57.0	507
2. Recognition of competencies	208	41.0	299	59.0	507
6. Logistical support	190	37.5	317	62.5	507
3. Protection	181	35.7	326	64.3	507
20. Optimism	175	34.5	332	65.5	507
1. Empathy	169	33.3	338	66.7	507
9. Professional competence for their family	131	25.8	376	74.2	507
7. Professional competence for their treatment	112	22.1	395	77.9	507

### 3.1 Relationship between mental health aspects and perceptions quality of the health services

**FIGURE 1:**
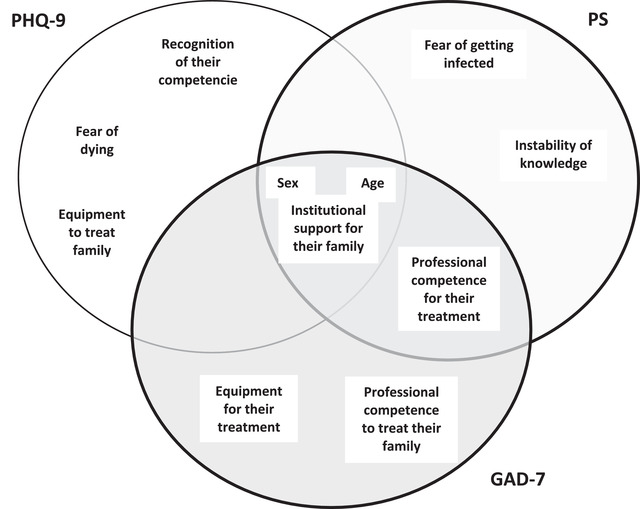
Representation of the relationship between PHQ‐9, PS and GAD‐7 scores, age, and sex and PQHS among noninfected HP. Heteroscedastic linear regression

The relationship between depression, anxiety, and stress scores and each PQHS item is shown in Table 3 (bivariate analysis), Table 4 (multivariate analysis), and Figure 1.

**TABLE 3:** Relationship between PHQ‐9, GAD‐7, and PS scores, age, and gender and PQHS among noninfected HP bivariate analysis

**Variables**	**PHQ‐9**			**GAD‐7**			**PS**		
	**rc**	**95% CI**	**p**	**rc**	**95% CI**	**p**	**rc**	**95% CI**	**p**
Age	−0.11	−0.14, −0.07	<.001	−0.86	−0.12, −0.05	<.001	−0.10	−0.14, −0.06	<.001
Gender	−2.14	−2.99, −1.28	<.001	−2.32	−3.16, −1.49	<.001	−2.84	−3.91, −1.76	<.001
1. Empathy	−1.55	−2.46, −0.65	.001	−1.19	−2.09, −0.29	.010	−0.60	−1.77, −0.56	.311
2. Recognition of their competencies	−1.19	−2.06, −0.32	.007	−0.054	−1.40, −0.33	.224	−0.58	−1.70, −0.54	.312
3. Protection	−1.26	−2.15, −0.36	.006	−0.80	−1.69, −0.09	.078	−0.84	−1.99, −0.31	.150
4. Personal early diagnosis	−1.09	−1.95, −0.23	.013	−0.82	−1.67, −0.03	.060	−1.25	−2.35, −0.15	.025
5. Family early diagnosis	−0.75	−1.64, −0.13	.095	−0.50	−1.38, −0.37	.260	−1.25	−2.38, −0.12	.030
6. Logistic support	−1.15	−2.03, −0.26	.011	−0.83	−1.71, −0.05	.064	−0.75	−1.90, −0.39	.198
7. Professional competence to treat	−2.44	−3.80, −1.08	<.001	−3.24	−4.58, −1.91	<.001	−3.73	−5.46, −2.00	<.001
8. Equipment to treat	−1.07	−2.11, −0.03	.043	−1.04	−2.07, −0.01	.046	−1.58	−2.90, −0.26	.019
9. Professional competence to treat their family	−1.22	−2.09, −0.36	.006	−1.26	−2.12, −0.41	.004	−1.32	−2.43, −0.21	.019
10. Equipment to treat their family	−1.48	−2.36, −0.61	.001	−1.45	−2.32, −0.58	.001	−1.79	−2.90, −0.67	.002
11. Fear of infection	−1.44	−2.42, −0.46	.004	−1.13	−2.10, −0.16	.023	−2.01	−3.25, −0.76	.002
12. Fear of infecting loved ones	−1.37	−3.01, −0.26	.100	−1.25	−2.87, −0.37	.131	−1.88	−3.46, −0 .72	.200
13. Fear of dying	−1.46	−2.32, −0.61	.001	−1.23	−2.08, −0.39	.004	−1.27	−2.37, −0.17	.023
14. Fear of a family member dying	−0.92	−1.87, −0.04	.061	−1.52	−2.46, −0.58	.002	−1.87	−3.08, −0.65	.003
15. Role change	−0.74	−1.62, −0.13	.097	−0.64	−1.50, −0.23	.150	−0.72	−1.83, −0.40	.210
16. Instability of knowledge	−1.29	−2.17, −0.42	.004	−1.35	−2.21, −0.48	.002	−2.58	−3.69, −1.48	<.001
17. Job burnout	−1.31	−2.39, −0.22	.018	−1.51	−2.58, −0.44	.006	−3.02	−4.39, −1.66	<.001
18. Concern for their family	−0.99	−1.92, −0.06	.037	−2.13	−3.03, −1.22	<.001	−2.67	−3.84, −1.50	<.001
19. Institutional support for their family	−2.49	−3.33, −1.64	<.001	−3.16	−398, −2.34	<.001	−3.62	−4.69, −2.55	<.001
20. Optimism	−0.76	−1.67, −0.14	.098	−0.82	−1.71, −0.77	.073	−1.24	−2.39, −0.09	.035

**TABLE 4:** Relationship between PHQ‐9, GAD‐7, and PS scores, age, and sex and PQHS among noninfected HP heteroscedastic linear regression

**Variables**	**PHQ‐9**			**GAD‐7**			**PS**		
	**rc**	**95% CI**	**p**	**rc**	**95% CI**	**p**	**rc**	**95% CI**	**p**
Age	−0.10	−0.13, −0.07	<.001	−0.07	−0.10, −0.04	<.001	−0.09	−0.12, −0.05	<.001
Gender	−1.34	−2.16, −0.52	<.001	−1.62	−2.43, −0.82	<.001	−1.51	−2.58, −0.44	.006
2. Recognition of their competencies	−1.62	−2.47, −0.78	<.001						
4. Personal early diagnosis							−1.24	−2.29, −0.20	.020
7. Professional competence to treat				−1.95	−3.21, −0.69	.002	−1.94	−3.60, −0.27	.022
8. Equipment to treat				−1.22	−2.25, −0.18	.021			
9. Professional competence to treat their family				−1.07	−1.92, −0.21	.015			
10. Equipment to treatment their family	−1.28	−2.13, −0.43	.003						
12. Fear of infection							−2.10	−3.30, −0.90	.001
13. Fear of dying	−1.14	−1.96, −0.32	.006						
16. Stability of knowledge							−1.25	−2.35, −0.14	.027
19. Institutional support for their family	−2.32	−3.16, −1.48	.001	0.40	−3.63, −2.04	<.001	−2.98	−4.08, −1.88	<.001

### 3.2 Depression

In the bivariate analysis, significant relationships were found between depression scores and all PQHS items, except for 3 items relating to family (“early family diagnosis,” “fear of infecting loved ones,” and “fear of a family member dying”) and the item “optimism.”

In the multivariate analysis, the factors related to levels of depression were younger age, female gender, lack of “recognition of skills,” lack of “equipment to treat family,” “fear of dying,” and lack of “institutional support for family”.

### 3.3 Anxiety

In the bivariate analysis, significant relationships were found between anxiety scores and most of the PQHS items, except for “recognition of competencies,” “protection,” “early personal diagnosis,” “early family diagnosis,” “logistical support,” and “optimism.”

In the multivariate analysis, the factors related to anxiety levels were younger age, female gender, lack of “professional competence for treatment,” lack of “equipment for treatment,” lack of “professional competence to treat family,” and lack of “institutional support for family.”

### 3.4 Perceived stress

In the bivariate analysis, significant relationships were found between the PS scores and most of the PQHS items, except for “empathy,” “recognition of competencies,” “protection,” “logistical support,” and “fear of infecting loved ones.”

In the multivariate analysis, the factors related to acute stress levels were younger age, female gender, lack of “early personal diagnosis,” lack of “professional competence for treatment,” “fear of infection,” lack of “instability of knowledge,” and lack of “institutional support for family.”

## 4 DISCUSSION

The results of this study indicate that there is a relationship between PQHS and the mental health of HP. In the bivariate analysis, significant relationships were found between anxiety, depression, and PS scores with most PQHS items. In the multivariate analysis, younger age and female gender were related to symptoms of anxiety, depression, and PS, all with statistical significance (*p* < .001) (Table 3). These findings are consistent with those of a study in Spain (Santamaría et al., 2021) in which women had higher levels of anxiety (*p* = .01) and stress (*p* = .03). Another study reported a relationship between anxiety, younger age and female sex (*p* = .001 for both variables) (Cag et al., 2021). In Ghana, stress was evaluated in 400 HP, with a higher prevalence in females (*p* < .001) (26). Finally, in Cyprus, the perception of lack of institutional support in the context of the pandemic predominated at younger ages (*p* < .001) and among females (*p* = .002) (Chatzittofis et al., 2021).

When we examined PQHS factors pertaining to the prevention and response of HS to the infection and/or death of workers and/or their family members through multivariate analysis, there was a clear association with anxiety, depression, and PS levels. The perception of HP regarding the lack of competencies of coworkers to care for them in case of infection was associated with anxiety and PS (*p* = .002 and *p* = .022, respectively). Additionally, the perception of lack of equipment to care for HP and their families in case of infection was associated with anxiety and depression (*p* = .021 and *p* = .003), respectively. Furthermore, symptoms of anxiety, depression and PS were related to the perception that there would be no support from the health institution if HP or their families were infected with COVID‐19 (*p* = .001, *p* < .001, and *p* < .001, respectively). These findings are consistent with the results of several studies, such as the one conducted in the USA (Cag et al., 2021) in which not having adequate protective equipment against COVID‐19 was associated with anxiety (*p* < .001). Another study in the United States (Obrenovic et al., 2021) found associations between perceptions of not having adequate protective equipment and fear of infection with COVID‐19 with anxiety and depression (*p* < .001 in both cases). A similar result was found in another study conducted with 1416 HP in 75 countries (Siddiqui et al., 2021); in that study, anxiety levels were related to insufficient protective equipment (*p* = .005). In another study, a survey was administered to 723 workers from 4 hospitals in the city of Tehran (Daneshvar et al., 2022), finding that HP’ fear of being infected with COVID‐19 or infecting their family and the uncertainty about whether they would receive support from HS was associated with anxiety. One study (Chatzittofis et al., 2021) showed that a poor perception of support from HS was associated with the self‐reported intensity of depression and PS (*p* < .001). Finally, a study conducted by the Pan American Health Organization (PAHO) with HP (Organización Panamericana de la Salud, 2022) found that the symptoms of depression were significantly associated with, among other perceptions, the concern of HP being infected themselves and infecting their relatives.

When exploring the PQHS related to the fear of infection, death of the worker and/or a family member due to working at a hospital, multivariate analysis revealed that the perception of becoming infected and dying was associated with depression (*p* = .006). Our findings are consistent with those reported in similar studies, i.e., there is an association between the fear of the HP being infected and depression (*p* < .001) (Young et al., 2021); the fear of infecting family members and depression (*p* < .001) (Obrenovic et al., 2021); and workers’ fear of personal and family infection and PS (*p* < .01) (Santamaría et al., 2021).

In this study, for PQHS, those items related the adequacy of institutional leadership and HP taking responsibility for themselves in how they work, expressed by the scores for recognition of their competencies and instability of knowledge about COVID‐19, were significantly associated with depression and PS (*p* < .001 and *p* = .027, respectively). Related studies have reported similar results. One study showed that depression was associated with work demands (*p* < .01) and anxiety when thinking about a family member at work (*p* < .001) (Young et al., 2021). Another study showed that being uniformed about SARS‐CoV‐2 was associated with anxiety (*p* = .005) (Cag et al., 2021), while in another study, such a lack of information was associated with depression and anxiety (*p* < .001, for both conditions) (Organización Panamericana de la Salud, 2022).

These findings clearly show that PQHS is a greater stressor in the daily work of HP, even more so than the health crisis generated by COVID‐19 or any other agent. Therefore, PQHS of HP should be continuously evaluated and considered a factor of change in the structure and dynamics of health institutions to minimize the risks of impacting the mental health of these workers (Organización Panamericana de la Salud, 2022; World Health Organization, 2021b). The stressor of inadequate institutional conditions for health care tasks, which leads to an unhealthy PQHS, will become a predominant factor in the deteriorating mental health of HP facing any health emergency (Siddiqui et al., 2021).

We propose that HP need to be able to express their concerns in a systematic and ongoing way about how HS are meeting their responsibilities of leadership, protection, and support of staff and their families during health emergencies such as the COVID‐19 pandemic. Only in this way can effective individual and group psychotherapeutic interventions be implemented consistently.

This study has several limitations. First, it was a cross‐sectional study based on an online questionnaire distributed to HP. Generalization of the conclusions is not possible because we did not have a means of approximating the entire population of HP. A longitudinal design is needed to provide definitive evidence of the resilience of health workers against mental health problems related to COVID‐19. Second, the participants were heterogeneous, and direct conclusions could not be applied to any particular professional group, whether they were doctors, nurses, midwives or others. Third, the participants were from Lima and the surrounding region; therefore, our findings cannot be generalized to the less affected regions of Peru or to professionals from other countries due to cultural differences and differences in health systems. Fourth, this study was based on a self‐administered questionnaire, and it was not possible to verify mental health problems through structured interviews. Fifth, this study did not differentiate between preexisting mental health symptoms and new symptoms related to COVID‐19 care.

## 5 CONCLUSIONS

There is a significant relationship between the mental health of HP who faced the COVID‐19 pandemic, expressed through the report of anxiety, depression and PS, and the female gender, younger age and PQHS. Symptoms of anxiety, depression and PS were associated with perceptions of low efficacy of the prevention and response mechanisms of HS regarding workers or their family members being infected or dying; the fear of HP or their family members being infected or dying because of working at a hospital; the lack of institutional leadership; and the negative impact on the work of HP.

## CONFLICT OF INTEREST

The authors declare no conflict of interest

### PEER REVIEW

The peer review history for this article is available at https://publons.com/publon/10.1002/brb3.2816

## Data Availability

Data available on request due to privacy/ethical restrictions. cd_value_code=text
